# *tert*-Butyl *N*-[*N*,*N*-bis­(2-chloro­ethyl)sulfamo­yl]-*N*-(2-chloro­ethyl)carbamate

**DOI:** 10.1107/S1600536809038185

**Published:** 2009-09-26

**Authors:** Achour Seridi, Hocine Akkari, Jean-Yves Winum, Patricia Bénard-Rocherullé, Mohamed Abdaoui

**Affiliations:** a Département des Sciences Fondamentales, Faculté des Sciences, Université du 20 Août 1955 – Skikda, Route d′El-Hadaïk, BP 26, 21000 Skikda, Algeria; b Institut des Biomolécules Max Mousseron, Ecole Nationale Supérieure de Chimie de Montpellier, 8, Rue de l’Ecole Normale, 34296 Montpellier Cedex, France; c Sciences Chimiques de Rennes (UMR CNRS 6226), Université de Rennes 1, Avenue du Général Leclerc, 35042 Rennes Cedex, France; d Laboratoire de Chimie Appliquée, Université du 8 Mai 1945 – Guelma, BP 401, 24000 Guelma, Algeria

## Abstract

The title compound, C_11_H_21_Cl_3_N_2_O_4_S, was produced as part of a development programme of a new synthetic route to chloro­ethyl­nitro­sosulfamides (CENS) with three chloro­ethyl moieties. These compounds possess structural features that confer potential biological activity and act as alkyl­ating agents. The packing is governed by four weak C—H⋯O inter­actions, forming an infinite three-dimensional network.

## Related literature

For the potential biological activity, pharmaceutical utility and cytotoxic activity of chloro­ethyl­nitro­sosulfamides, see: Abdaoui *et al.* (1996 ▶, 2000 ▶); Dokhane *et al.* (2002 ▶); Galešić *et al.* (1987 ▶); Gnewuch & Sosnovsky (1997 ▶); Ishiguro *et al.* (2006 ▶); Jonnalagadda *et al.* (2007 ▶); Passagne *et al.* (2003 ▶); Seridi *et al.* (2006 ▶); Skinner & Scharts (1972 ▶); Voutsinas *et al.* (1993 ▶); Winum *et al.* (2003 ▶). For the synthetic procedure, see: Mitsunobu (1981 ▶).
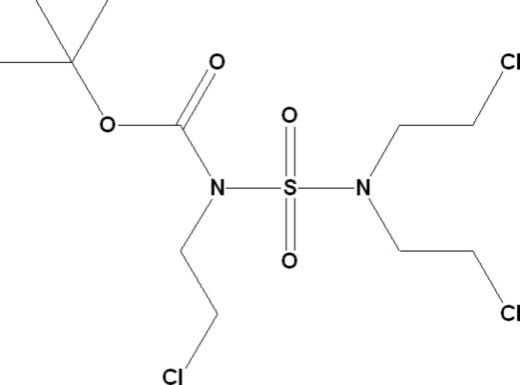


## Experimental

### 

#### Crystal data

C_11_H_21_Cl_3_N_2_O_4_S
*M* *_r_* = 383.71
Monoclinic, 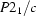

*a* = 9.6132 (5) Å
*b* = 17.1282 (9) Å
*c* = 10.6763 (5) Å
β = 93.868 (3)°
*V* = 1753.92 (15) Å^3^
*Z* = 4
Mo *K*α radiation
μ = 0.66 mm^−1^
*T* = 100 K
0.15 × 0.12 × 0.1 mm

#### Data collection

Bruker APEXII diffractometer
Absorption correction: multi-scan (*SADABS*; Sheldrick, 2002 ▶) *T*_min_ = 0.862, *T*_max_ = 0.937
17775 measured reflections
3982 independent reflections
3662 reflections with *I* > 2σ(*I*)
*R*_int_ = 0.037

#### Refinement

*R*[*F* ^2^ > 2σ(*F* ^2^)] = 0.026
*wR*(*F* ^2^) = 0.068
*S* = 1.03
3982 reflections
193 parameters
H-atom parameters constrained
Δρ_max_ = 0.37 e Å^−3^
Δρ_min_ = −0.39 e Å^−3^

### 

Data collection: *SMART* (Bruker, 2006 ▶); cell refinement: *SAINT* (Bruker, 2006 ▶); data reduction: *SAINT*; program(s) used to solve structure: *SIR97* (Altomare *et al.*, 1999 ▶); program(s) used to refine structure: *SHELXL97* (Sheldrick, 2008 ▶); molecular graphics: *ORTEP-3 for Windows* (Farrugia, 1997 ▶); software used to prepare material for publication: *WinGX* (Farrugia, 1999 ▶).

## Supplementary Material

Crystal structure: contains datablocks global, I. DOI: 10.1107/S1600536809038185/dn2489sup1.cif

Structure factors: contains datablocks I. DOI: 10.1107/S1600536809038185/dn2489Isup2.hkl

Additional supplementary materials:  crystallographic information; 3D view; checkCIF report

## Figures and Tables

**Table 1:** Hydrogen-bond geometry (Å, °)

*D*—H⋯*A*	*D*—H	H⋯*A*	*D*⋯*A*	*D*—H⋯*A*
C2—H2*B*⋯O1^i^	0.97	2.59	3.5465 (16)	167
C8—H8*B*⋯O1^i^	0.97	2.58	3.5047 (17)	159
C9—H91⋯O3^ii^	0.97	2.39	3.3156 (17)	160
C11—H11*B*⋯O2^ii^	0.97	2.44	3.0428 (16)	120

Symmetry codes: (i) 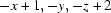; (ii) 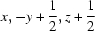.

**Fig. 1.**
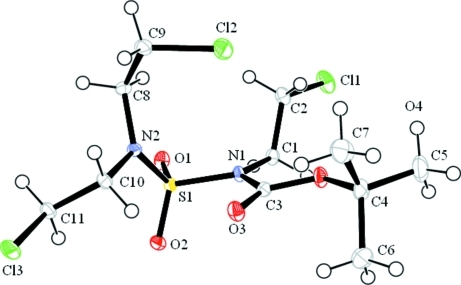
(Farrugia, 1997) The molecule of the title compound in the crystal. Ellipsoids correspond to 50% probability levels and H atoms are shown as small spheres of arbitrary radii.

**Fig. 2.**
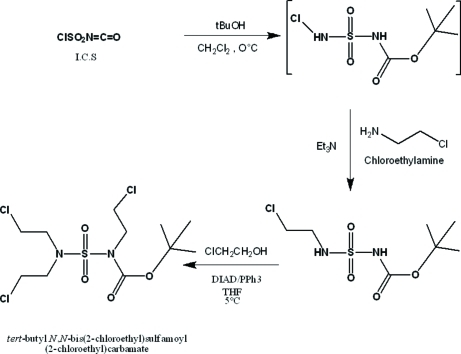
Synthesis of the title compound.

## supplementary crystallographic information

### Comment

Compounds with one or more *N*-(2-chloroethyl) moieties show many pharmacological activities (Galešić *et al.*, 1987). They are cytotoxic (Ishiguro *et al.*, 2006), mutagenic (Voutsinas *et al.*, 1993), and immuno-suppressive (Skinner & Scharts, 1972). Many of them include Mechlorethamine, Chlorambucil, Melphalan,Cyclophosmamide, Ifosfamide, are used for the treatment of wide variety of cancers (Jonnalagadda *et al.*, 2007). Among others, *N*-(2-chloroethyl) nitrososulfamides (CENS) are promising antitumoral agents which have been developed as new family of alkylating agents structurally related to 2-chloroethylnitrosoureas (CENU) (Abdaoui *et al.*, 1996). A certain number of these derivatives exhibited interesting cytotoxic activity and among them, some prouved to be considerably more potent than the parent nitrosourea (Abdaoui *et al.*, 2000; Gnewuch & Sosnovsky, 1997; Passagne *et al.*, 2003; Seridi *et al.*, 2006; Winum *et al.*, 2003).

In order to extend our knowledge about such sulfamides derivatives with three *N*-(2-chloroethyl) moieties the crystal structure of the title compound is presented.

In all essential details, the molecular geometry in terms of bond distances and angles is in good agreement with related structure (Dokhane *et al.* 2002). In the molecular geometry (Fig.1), the sulfamide moiety N1—S—N2 exhibit an asymmetry of S—N bond distance, with values of 1.688 (1) and 1.615 (1) Å respectively. The molecules are linked by four C—H···O intermolecular interactions involving sulfonamide (oxygen atoms O1 and O2) and carbonyl (oxygen atom O3) functions (table 1). Thus, these interactions lead to an infinite three-dimensional network.

### Experimental

The synthetic pathway used for the preparation of the title compound is outlined in Fig. 2. First the formation of *tert*-butyl*N*-(2-chloroethyl)sulfamoylcarbamate which is performed in dried dichloromethane with successive addition of tBuOH, and Chloroethylamine/TEA into CSI. After purification, the carbamate was recovered at (yield 80%). The second step is carried out according to the Mitsunobu procedure (Mitsunobu, 1981) in anhydrous THF as a solvent. The mixture of DEAD (diethyl azodicarboxylate) and *tert*-butyl*N*-(2-chloroethyl)sulfamoylcarbamate is added to a solution of excess of chloroethanol and PPh3. The product was recrystallized in pure ethanol.

### Refinement

H atoms bonded to C atoms were positioned geometrically and refined isotropically using a riding model (including free rotation about the ethanol C—C bond), with C—H = 0.97 Å (methylene) or 0.96Å (methyl) and with *U*_iso_(H) = 1.2 (1.5 for methyl groups) times *U*_eq_(C).

### Crystal data

**Table:**

C_11_H_21_Cl_3_N_2_O_4_S	*F*(000) = 800
*M**_r_* = 383.71	*D*_x_ = 1.453 Mg m^−^^3^
Monoclinic, *P*2_1_/*c*	Mo *K*α radiation, λ = 0.71073 Å
Hall symbol: -P 2ybc	Cell parameters from 9788 reflections
*a* = 9.6132 (5) Å	θ = 2.4–27.4°
*b* = 17.1282 (9) Å	µ = 0.66 mm^−^^1^
*c* = 10.6763 (5) Å	*T* = 100 K
β = 93.868 (3)°	Prism, colourless
*V* = 1753.92 (15) Å^3^	0.15 × 0.12 × 0.1 mm
*Z* = 4	

### Data collection

**Table:**

Bruker APEXII diffractometer	3982 independent reflections
Radiation source: APEXII, Bruker-AXS	3662 reflections with *I* > 2σ(*I*)
graphite	*R*_int_ = 0.037
CCD rotation images, thick slices scans	θ_max_ = 27.4°, θ_min_ = 2.1°
Absorption correction: multi-scan (*SADABS*; Sheldrick, 2002)	*h* = −12→11
*T*_min_ = 0.862, *T*_max_ = 0.937	*k* = −22→21
17775 measured reflections	*l* = −13→13

### Refinement

**Table:**

Refinement on *F*^2^	Primary atom site location: structure-invariant direct methods
Least-squares matrix: full	Secondary atom site location: difference Fourier map
*R*[*F*^2^ > 2σ(*F*^2^)] = 0.026	Hydrogen site location: inferred from neighbouring sites
*wR*(*F*^2^) = 0.068	H-atom parameters constrained
*S* = 1.03	*w* = 1/[σ^2^(*F*_o_^2^) + (0.0272*P*)^2^ + 0.8873*P*] where *P* = (*F*_o_^2^ + 2*F*_c_^2^)/3
3982 reflections	(Δ/σ)_max_ = 0.001
193 parameters	Δρ_max_ = 0.37 e Å^−^^3^
0 restraints	Δρ_min_ = −0.39 e Å^−^^3^

### Special details

**Table:**

Geometry. All esds (except the esd in the dihedral angle between two l.s. planes) are estimated using the full covariance matrix. The cell esds are taken into account individually in the estimation of esds in distances, angles and torsion angles; correlations between esds in cell parameters are only used when they are defined by crystal symmetry. An approximate (isotropic) treatment of cell esds is used for estimating esds involving l.s. planes.
Refinement. Refinement of *F*^2^ against ALL reflections. The weighted *R*-factor *wR* and goodness of fit *S* are based on *F*^2^, conventional *R*-factors *R* are based on *F*, with *F* set to zero for negative *F*^2^. The threshold expression of *F*^2^ > σ(*F*^2^) is used only for calculating *R*-factors(gt) *etc*. and is not relevant to the choice of reflections for refinement. *R*-factors based on *F*^2^ are statistically about twice as large as those based on *F*, and *R*- factors based on ALL data will be even larger.

### Fractional atomic coordinates and isotropic or equivalent isotropic displacement parameters (Å^2^)

**Table:**

	*x*	*y*	*z*	*U*_iso_*/*U*_eq_	
C1	0.30088 (13)	−0.00247 (8)	0.74767 (12)	0.0142 (3)	
H1A	0.3977	−0.0155	0.7375	0.017*	
H1B	0.2483	−0.0120	0.6684	0.017*	
C2	0.24504 (14)	−0.05402 (8)	0.84875 (13)	0.0166 (3)	
H2A	0.1511	−0.0381	0.8649	0.020*	
H2B	0.3033	−0.0489	0.9261	0.020*	
C3	0.16945 (13)	0.12486 (8)	0.74993 (12)	0.0141 (3)	
C4	−0.07329 (14)	0.11154 (9)	0.66503 (14)	0.0212 (3)	
C5	−0.15279 (16)	0.03860 (10)	0.62057 (17)	0.0332 (4)	
H5A	−0.1090	0.0162	0.5508	0.050*	
H5B	−0.2473	0.0523	0.5950	0.050*	
H5C	−0.1521	0.0014	0.6878	0.050*	
C6	−0.05995 (16)	0.16890 (10)	0.55745 (15)	0.0281 (3)	
H6A	−0.0128	0.2151	0.5885	0.042*	
H6B	−0.1511	0.1825	0.5220	0.042*	
H6C	−0.0075	0.1452	0.4941	0.042*	
C7	−0.13690 (15)	0.14725 (10)	0.77813 (15)	0.0285 (3)	
H7A	−0.1375	0.1092	0.8442	0.043*	
H7B	−0.2307	0.1635	0.7550	0.043*	
H7C	−0.0827	0.1916	0.8068	0.043*	
C8	0.38802 (13)	0.13068 (8)	1.08849 (12)	0.0150 (3)	
H8A	0.4597	0.1523	1.1468	0.018*	
H8B	0.4115	0.0765	1.0741	0.018*	
C9	0.24946 (14)	0.13378 (8)	1.14857 (13)	0.0184 (3)	
H91	0.2239	0.1879	1.1609	0.022*	
H92	0.2594	0.1090	1.2304	0.022*	
C10	0.36795 (13)	0.25857 (8)	0.96777 (12)	0.0139 (3)	
H10A	0.3144	0.2731	1.0379	0.017*	
H10B	0.3146	0.2733	0.8910	0.017*	
C11	0.50586 (14)	0.30294 (8)	0.97652 (12)	0.0158 (3)	
H11A	0.5593	0.2904	0.9053	0.019*	
H11B	0.5605	0.2888	1.0528	0.019*	
Cl1	0.24477 (4)	−0.15386 (2)	0.79617 (4)	0.02567 (9)	
Cl2	0.11302 (3)	0.08604 (2)	1.05440 (3)	0.02616 (10)	
Cl3	0.46514 (4)	0.40555 (2)	0.97723 (3)	0.02271 (9)	
N1	0.29004 (10)	0.08109 (6)	0.78128 (10)	0.0123 (2)	
N2	0.38924 (11)	0.17329 (6)	0.96878 (9)	0.0121 (2)	
O1	0.52796 (9)	0.06756 (6)	0.87615 (9)	0.01627 (19)	
O2	0.46793 (9)	0.18884 (6)	0.75688 (8)	0.0161 (2)	
O3	0.16339 (9)	0.19465 (6)	0.76460 (9)	0.0177 (2)	
O4	0.06748 (9)	0.07794 (6)	0.70365 (9)	0.0181 (2)	
S1	0.43157 (3)	0.129314 (19)	0.84310 (3)	0.01123 (8)	

### Atomic displacement parameters (Å^2^)

**Table:**

	*U*^11^	*U*^22^	*U*^33^	*U*^12^	*U*^13^	*U*^23^
C1	0.0159 (6)	0.0112 (7)	0.0153 (6)	0.0002 (5)	−0.0004 (4)	−0.0037 (5)
C2	0.0169 (6)	0.0110 (7)	0.0219 (7)	−0.0006 (5)	0.0011 (5)	−0.0016 (5)
C3	0.0131 (6)	0.0153 (7)	0.0136 (6)	−0.0015 (5)	−0.0015 (4)	0.0027 (5)
C4	0.0122 (6)	0.0221 (8)	0.0283 (7)	−0.0004 (5)	−0.0076 (5)	0.0057 (6)
C5	0.0238 (8)	0.0296 (9)	0.0438 (10)	−0.0088 (6)	−0.0159 (7)	0.0055 (7)
C6	0.0227 (7)	0.0311 (9)	0.0292 (8)	−0.0003 (6)	−0.0074 (6)	0.0102 (7)
C7	0.0161 (7)	0.0343 (9)	0.0347 (9)	0.0020 (6)	0.0001 (6)	0.0067 (7)
C8	0.0183 (6)	0.0143 (7)	0.0120 (6)	−0.0011 (5)	−0.0008 (5)	0.0028 (5)
C9	0.0248 (7)	0.0161 (7)	0.0149 (6)	−0.0041 (5)	0.0052 (5)	−0.0016 (5)
C10	0.0165 (6)	0.0094 (6)	0.0158 (6)	−0.0009 (5)	0.0008 (5)	0.0000 (5)
C11	0.0197 (6)	0.0114 (7)	0.0166 (6)	−0.0031 (5)	0.0027 (5)	−0.0008 (5)
Cl1	0.02584 (18)	0.01139 (18)	0.0396 (2)	−0.00249 (13)	0.00118 (14)	−0.00252 (14)
Cl2	0.01773 (16)	0.0387 (2)	0.02252 (18)	−0.00801 (14)	0.00518 (12)	−0.00232 (15)
Cl3	0.03418 (19)	0.01075 (17)	0.02329 (18)	−0.00579 (13)	0.00268 (13)	0.00063 (12)
N1	0.0116 (5)	0.0097 (6)	0.0153 (5)	−0.0015 (4)	−0.0014 (4)	−0.0013 (4)
N2	0.0158 (5)	0.0093 (5)	0.0112 (5)	−0.0010 (4)	0.0014 (4)	−0.0002 (4)
O1	0.0130 (4)	0.0164 (5)	0.0190 (5)	0.0027 (4)	−0.0014 (3)	−0.0026 (4)
O2	0.0182 (4)	0.0162 (5)	0.0139 (4)	−0.0048 (4)	0.0031 (3)	0.0001 (4)
O3	0.0164 (4)	0.0112 (5)	0.0247 (5)	0.0001 (3)	−0.0037 (4)	0.0019 (4)
O4	0.0138 (4)	0.0142 (5)	0.0251 (5)	−0.0013 (4)	−0.0071 (4)	0.0017 (4)
S1	0.01039 (14)	0.01148 (17)	0.01178 (15)	−0.00120 (11)	0.00045 (10)	−0.00080 (11)

### Geometric parameters (Å, °)

**Table:**

C1—N1	1.4809 (17)	C7—H7B	0.9600
C1—C2	1.5201 (18)	C7—H7C	0.9600
C1—H1A	0.9700	C8—N2	1.4725 (16)
C1—H1B	0.9700	C8—C9	1.5178 (18)
C2—Cl1	1.7997 (14)	C8—H8A	0.9700
C2—H2A	0.9700	C8—H8B	0.9700
C2—H2B	0.9700	C9—Cl2	1.7950 (14)
C3—O3	1.2074 (17)	C9—H91	0.9700
C3—O4	1.3364 (15)	C9—H92	0.9700
C3—N1	1.4020 (16)	C10—N2	1.4749 (17)
C4—O4	1.5024 (15)	C10—C11	1.5255 (17)
C4—C7	1.519 (2)	C10—H10A	0.9700
C4—C6	1.523 (2)	C10—H10B	0.9700
C4—C5	1.524 (2)	C11—Cl3	1.8007 (14)
C5—H5A	0.9600	C11—H11A	0.9700
C5—H5B	0.9600	C11—H11B	0.9700
C5—H5C	0.9600	N1—S1	1.6875 (10)
C6—H6A	0.9600	N2—S1	1.6147 (11)
C6—H6B	0.9600	O1—S1	1.4345 (10)
C6—H6C	0.9600	O2—S1	1.4326 (10)
C7—H7A	0.9600		
			
N1—C1—C2	110.82 (10)	H7B—C7—H7C	109.5
N1—C1—H1A	109.5	N2—C8—C9	114.08 (11)
C2—C1—H1A	109.5	N2—C8—H8A	108.7
N1—C1—H1B	109.5	C9—C8—H8A	108.7
C2—C1—H1B	109.5	N2—C8—H8B	108.7
H1A—C1—H1B	108.1	C9—C8—H8B	108.7
C1—C2—Cl1	108.88 (9)	H8A—C8—H8B	107.6
C1—C2—H2A	109.9	C8—C9—Cl2	112.10 (9)
Cl1—C2—H2A	109.9	C8—C9—H91	109.2
C1—C2—H2B	109.9	Cl2—C9—H91	109.2
Cl1—C2—H2B	109.9	C8—C9—H92	109.2
H2A—C2—H2B	108.3	Cl2—C9—H92	109.2
O3—C3—O4	127.07 (12)	H91—C9—H92	107.9
O3—C3—N1	123.01 (11)	N2—C10—C11	111.92 (10)
O4—C3—N1	109.92 (11)	N2—C10—H10A	109.2
O4—C4—C7	109.86 (11)	C11—C10—H10A	109.2
O4—C4—C6	109.51 (11)	N2—C10—H10B	109.2
C7—C4—C6	113.49 (13)	C11—C10—H10B	109.2
O4—C4—C5	101.24 (11)	H10A—C10—H10B	107.9
C7—C4—C5	110.95 (13)	C10—C11—Cl3	107.35 (9)
C6—C4—C5	111.09 (13)	C10—C11—H11A	110.2
C4—C5—H5A	109.5	Cl3—C11—H11A	110.2
C4—C5—H5B	109.5	C10—C11—H11B	110.2
H5A—C5—H5B	109.5	Cl3—C11—H11B	110.2
C4—C5—H5C	109.5	H11A—C11—H11B	108.5
H5A—C5—H5C	109.5	C3—N1—C1	121.97 (10)
H5B—C5—H5C	109.5	C3—N1—S1	117.61 (9)
C4—C6—H6A	109.5	C1—N1—S1	119.98 (8)
C4—C6—H6B	109.5	C8—N2—C10	119.21 (10)
H6A—C6—H6B	109.5	C8—N2—S1	120.48 (9)
C4—C6—H6C	109.5	C10—N2—S1	119.90 (8)
H6A—C6—H6C	109.5	C3—O4—C4	119.72 (11)
H6B—C6—H6C	109.5	O2—S1—O1	120.08 (6)
C4—C7—H7A	109.5	O2—S1—N2	106.74 (6)
C4—C7—H7B	109.5	O1—S1—N2	109.53 (6)
H7A—C7—H7B	109.5	O2—S1—N1	108.80 (5)
C4—C7—H7C	109.5	O1—S1—N1	103.05 (5)
H7A—C7—H7C	109.5	N2—S1—N1	108.16 (5)

### Hydrogen-bond geometry (Å, °)

**Table:**

*D*—H···*A*	*D*—H	H···*A*	*D*···*A*	*D*—H···*A*
C2—H2B···O1^i^	0.97	2.59	3.5465 (16)	167
C8—H8B···O1^i^	0.97	2.58	3.5047 (17)	159
C9—H91···O3^ii^	0.97	2.39	3.3156 (17)	160
C11—H11B···O2^ii^	0.97	2.44	3.0428 (16)	120

Symmetry codes: (i) −*x*+1, −*y*, −*z*+2; (ii) *x*, −*y*+1/2, *z*+1/2.
